# Nomogram and a predictive model for postoperative hemorrhage in preoperative patients of laparoscopic pancreaticoduodectomy

**DOI:** 10.1038/s41598-021-94387-y

**Published:** 2021-07-20

**Authors:** Dongrui Li, Chengxu Du, Jiansheng Zhang, Zhongqiang Xing, Jianhua Liu

**Affiliations:** Department of Hepatobiliary Surgery, The Second Hospital of Hebei Medical University, 215 Heping West Road, Shijiazhuang, 050000 Hebei China

**Keywords:** Medical research, Risk factors, Signs and symptoms

## Abstract

To develop a predictive model and a nomogram for predicting postoperative hemorrhage in preoperative patients undergoing laparoscopic pancreaticoduodenectomy (LPD). A total of 409 LPD patients that underwent LPD by the same surgical team between January 2014 and December 2020 were included as the training cohort. The preoperative data of patients were statistically compared and analyzed for exploring factors correlated with postoperative hemorrhage. The predictive model was developed by multivariate logistic regression and stepwise (stepAIC) selection. A nomogram based on the predictive model was developed. The discriminatory ability of the predictive model was validated using the receiver operating characteristic (ROC) curve and leave-one-out method. The statistical analysis was performed using R 3.5.1 (www.r-project.org). The predictive model including the risk-associated factors of postoperative hemorrhage was as follows: 2.695843 − 0.63056 × (Jaundice = 1) − 1.08368 × (DM = 1) − 2.10445 × (Hepatitis = 1) + 1.152354 × (Pancreatic tumor = 1) + 1.071354 × (Bile duct tumor = 1) − 0.01185 × CA125 − 0.04929 × TT − 0.08826 × APTT + 26.03383 × INR − 1.9442 × PT + 1.979563 × WBC − 2.26868 × NEU − 2.0789 × LYM − 0.02038 × CREA + 0.00459 × AST. A practical nomogram based on the model was obtained. The internal validation of ROC curve was statistically significant (AUC = 0.7758). The validation by leave-one-out method showed that the accuracy of the model and the F measure was 0.887 and 0.939, respectively. The predictive model and nomogram based on the preoperative data of patients undergoing LPD can be useful for predicting the risk degree of postoperative hemorrhage.

## Introduction

Laparoscopic surgeries with minimal invasions have become possible because of the new advancements in laparoscopic technology and instruments in the past decade. Laparoscopic pancreaticoduodenectomy (LPD) is widely used for treating periampullary tumors nowadays^1,2^. Retrospective or prospective comparative studies pertaining to the differences in the clinical outcomes of patients that underwent LPD and that underwent open pancreaticoduodenectomy (OPD) reported that LPD is associated with shorter hospital stay^3–5^, lesser hospitalization cost^5,6^ and similar short-term outcomes and long-term survival^2,4,7–10^ compared with those of OPD. Similar to OPD, the morbidity and mortality of patients that underwent LPD is associated with postoperative complications, including postoperative pancreatic fistula, delayed gastric emptying, and postoperative hemorrhage^11,12^. Surgeons from various countries try to reduce the incidence of severe complications of LPD by modifying surgical procedures^13–16^. However, only a few studies have tried to predict the severe complications of LPD. Postoperative hemorrhage is a relatively frequent complication of LPD^17^, and is mainly induced by the formation of pancreatic fistula. Postoperative hemorrhage often requires reoperation or interventional embolization, which may secondly trigger complications involving perioperative death. There for. in this study, we aimed to develop a predictive model and a nomogram that can predict postoperative hemorrhage in preoperative patients undergoing LPD.

## Methods

### Patients

A total of 409 patients who underwent successful LPD without unexpected events by the same surgical team in the Department of Hepatobiliary Surgery of the Second Hospital of Hebei Medical University between January 2014 and December 2020 were included in this study as the training cohort. The inclusion criteria for the training cohort were the absence of (1) metastasis in other organs, (2) vascular invasion, (3) coexisting critical diseases, and the presence of comprehensive preoperative data. The preoperative data included general conditions (age, sex and BMI), symptoms before admission (jaundice, abdominal pain and fever), coexisting medical conditions (high blood pressure (HBP), coronary heart disease (CHD), diabetes mellitus (DM), pancreatitis, hepatitis and previous history of operation), preoperative treatment (bile duct drainage), tumor location (duodenum, bile duct, pancreas) and blood tests results (CA125, CA199, TT, fibrinogen (Fib), activated partial thromboplastin time (APTT), INR, PT, WBC, NEU, LYM, red blood cells (RBC), hemoglobin (HGB), platelet (PLT), total bilirubin (TBIL), albumin (ALB), alanine aminotransferase (ALT), AST, γ-glutamyl transferase (GGT), CREA.There were 173 females and 236 males with a mean age of 62 years. Among those, 43 patients developed postoperative hemorrhage. In this study, postoperative hemorrhage was defined as gastrointestinal hemorrhage or intra-abdominal hemorrhage and need of immediate reoperation or interventional embolization. The characteristics of the included patients are summarized in Table 1.

**Table 1:** The statistical characteristics of the perioperative data in the training cohort.

Variables	Hemorrhage	No hemorrhage	P_Comparison_	z value	P_Multivariate logistic model_
	n = 43,10.51%	n = 366,89.49%			
**General conditions**					
Age (years)	60.42 ± 10.56	58.81 ± 11.08	0.837	− 0.010	0.664
Sex (M/F)	26/17	210/156	0.698	− 0.411	0.681
BMI	24.09 ± 3.63	23.30 ± 3.74	0.699	− 0.992	0.321
**Sympotoms before admission**					
Jaudice (Y/N)	25/18	193/173	0.501	0.690	0.490
Abdominal pain (Y/N)	16/27	142/224	0.840	0.210	0.833
Fever (Y/N)	2/41	18/348	1.000	0.379	0.705
**Coexisting medical conditions**					
HPB (Y/N)	15/28	118/248	0.726	− 0.003	0.998
CHD (Y/N)	4/39	22/344	0.613	0.181	0.856
DM (Y/N)	11/32	51/315	0.044	− 2.198	0.028
Pancreatitis (Y/N)	2/41	15/351	1.000	− 0.209	0.835
Hepatitis (Y/N)	5/38	39/327	0.002	− 3.104	0.002
Previous surgical history (Y/N)	5/38	39/327	1.000	0.457	0.647
**Preoperative treatment**					
Cholangial drainage (Y/N)	19/24	141/225	0.472	0.450	0.652
**Tumor location**					
Duodenum (Y/N)	22/21	120/246	0.017	0.014	0.989
Bile duct (Y/N)	8/35	77/289	0.710	0.015	0.988
Pancreas (Y/N)	13/30	170/196	0.043	0.015	0.988
**Blood tests (median)**					
CA125 (U/mL)	10.82	13.00	0.119	− 2.281	0.023
CA199 (U/mL)	66.87	63.00	0.724	− 0.261	0.794
TT (s)	13.80	13.90	0.250	− 1.503	0.133
Fib (g/L)	4.05	3.86	0.803	0.547	0.584
APTT (s)	31.60	30.25	0.056	− 2.007	0.038
INR	0.95	0.98	0.026	1.574	0.115
PT (s)	10.60	10.90	0.049	− 1.354	0.176
WBC (10^9^/L)	6.60	6.20	0.300	2.382	0.017
NEU (10^9^/L)	4.40	4.02	0.209	− 2.501	0.012
LYM (10^9^/L)	1.50	1.45	0.816	− 2.267	0.023
RBC (10^12^/L)	3.98	4.08	0.363	1.444	0.149
HGB (g/L)	125.00	126.00	0.910	− 1.329	0.184
PLT (10^9^/L)	243.00	231.00	0.989	0.221	0.825
TBIL (μmol/L)	54.00	42.98	0.416	− 0.708	0.479
ALB (g/L)	37.10	38.15	0.467	0.377	0.760
ALT (U/L)	69.00	82.25	0.400	− 1.060	0.289
AST (U/L)	42.70	49.70	0.188	1.435	0.151
GGT (U/L)	279.00	273.00	0.499	0.625	0.532
CREA (μmol/L)	57.00	57.80	0.350	− 1.546	0.122

P_Comparison_: P value of the statistical comparison between the hemorrhage group and no hemorrhage group;

z value: The multivariate logistic regression analysis was performed to develop a full-variable logistic model.

P_Multivariate analysis_: P value of the multivariate logistic regression analysis of the risk-associated factors of postoperative hemorrhage.

*BMI* body mass index, *HPB* high blood pressure, *CHD* coronary heart disease, *DM* diabetes mellitus.

### Procedure and main steps of LPD

The resection of the specimen was performed in the following order. (1) The gastrocolic ligament was dissected and the duodenum was mobilized by performing the Kocher maneuver. (2) The right gastroepiploic and pancreaticoduodenal inferior vessels were dissected, ligated, and transected. (3) The distal stomach 2–3 cm from the pylorus was transected. (4) A tunnel was created between the pancreatic neck and the superior mesenteric vein (SMV) or portal vein at the inferior border of the pancreas. (5) The jejunum was exposed through the Riolan avascular area on the left of the SMV, and transected 15–20 cm distal from the Treitz ligament. (6) Lymphadenectomy of the hepatoduodenal ligament was performed and the gastroduodenal artery was transected. (7) The jejunum and duodenum were completely mobilized from left to right to expose major vasculatures. (8) The pancreas neck was transected. (9) Cholecystectomy was performed and the common hepatic duct was transected. (10). The inferior vena cava and the left renal vein were exposed by performing the Kocher maneuver. (11) The uncinate process was separated from the SMV. (12) Lymphadenectomy was performed, including the lymph node stations of 5, 6, 8a, 13a, 13b, 14a, 14b, 17a, and 17b. (13) The specimen placed in a retrieval bag was extracted through a 5-cm upper abdominal incision.

Reconstruction was performed as follows. (1) Pancreatojejunostomy: a two-layer duct-to-mucosa anastomosis was performed. (2) Choledochojejunostomy: an end-to-side with approximately 10 cm distal to the anastomosis of pancreatojejunostomy was performed. (3) Gastrojejunostomy: ante colic gastrojejunostomy was performed 40–45 cm downstream from the choledochojejunostomy.

All the LPD surgeries were performed by the same surgical team led by Professor Liu Jianhua, who was the operator. Ultrasonic Shears and linear staplers were used in the procedures, and the vessels were ligated using hemolock clips.

### Statistical analysis

The preoperative data of 409 patients who underwent LPD were retrospectively analyzed. The data were divided into the following two groups: with postoperative hemorrhage and without postoperative hemorrhage. Normality tests were performed for measurement data of each group. Between the two groups, the Chi-square tests were performed for the variables of sex, jaundice, abdominal pain, fever, HBP, CHD, DM, pancreatitis, hepatitis, previous history of operation, and different tumor locations. The student’s t tests were performed for variables of age and BMI. The Wilcoxon rank-sum tests were performed for the variables of blood test results. All the tests were two tailed, and a P value of less than 0.05 was considered statistically significant.

The predictive model was developed by performing multivariate logistic regression analysis and stepwise (stepAIC) selection. The data were first analyzed by multivariate binary logistic regression analysis to reveal the factors correlated with postoperative hemorrhage and to develop a full-variable predictive model. Stepwise (stepAIC) selection was then performed to obtain the best predictive model. A nomogram based on the predictive model was developed. The discriminatory ability of the predictive model was validated using the receiver operating characteristic (ROC) curve and leave-one-out method. The statistical analyses were performed using R 3.5.1(www.r-project.org).

This study was performed in accordance with the relevant guidelines and regulations and was approved by the Research Ethics Committee of the Second Hospital of Hebei Medical University. Informed consent was obtained from all the study subjects.

## Results

### Risk-associated factors of postoperative hemorrhage

Among the 409 patients, 43 developed postoperative hemorrhage with an incidence of 10.51%. The statistical comparative tests showed statistically significant differences in the coexisting medical conditions of DM (P = 0.003) and hepatitis (P = 0.004), tumors located in pancreas (P = 0.043) and duodenum (P = 0.017), and the coagulation function blood tests of INR (P = 0.026) and PT (P = 0.049) between the hemorrhage group and the no-hemorrhage group. Risk-associated factors of postoperative hemorrhage identified by multivariate logistic regression analysis in a full-variable predictive model were as follows: the coexisting medical conditions of DM (P = 0.028) and hepatitis (P = 0.002), the tumor marker of CA125 level (P = 0.023), the coagulation function blood tests of APTT (P = 0.038), blood routine examination of WBC (P = 0.017), NEU (P = 0.012), and LYM (P = 0.023). The statistical characteristics of the perioperative data of the patients in the training cohort are summarized in Table 1.

### Predictive model and nomogram for postoperative hemorrhage

**Figure 1:**
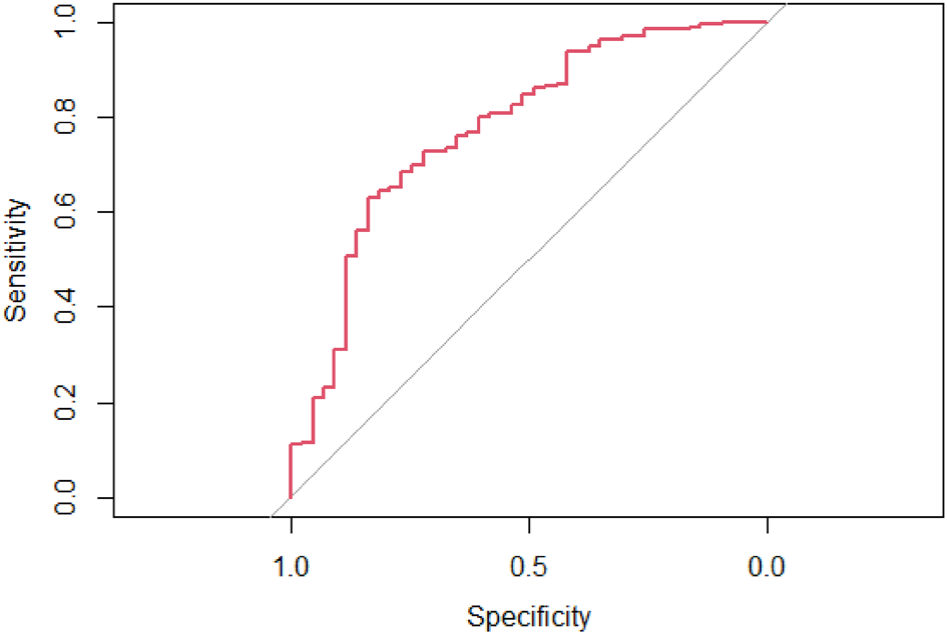
The ROC curve of the predictive model of postoperative hemorrhage of LPD.

**Figure 2:**
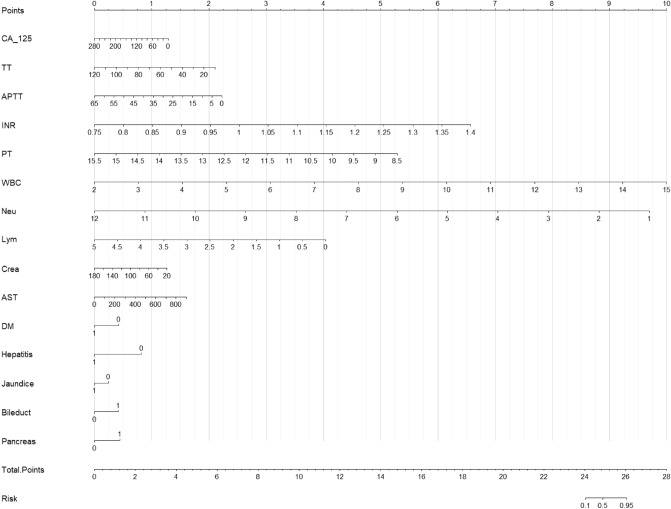
Predictive nomogram for postoperative hemorrhage of LPD.

The predictive model was developed by performing the multivariate logistic regression analysis. The best model was obtained by stepwise (stepAIC) selection. The risk-associated variables in the predictive model of postoperative hemorrhage included the symptom of jaundice (P = 0.005, 97.5% CI 0.236–1.165), coexisting medical conditions of DM (P = 0.028, 97.5% CI 0.147–0.807) and hepatitis (P = 0.002,97.5% CI 0.032–0.488), the tumors located in pancreas (P = 0.005, 97.5% CI 1.442–7.278) and bile duct (P = 0.033, 97.5% CI 1.136–8.338), and blood tests of CA125 level (P = 0.039, 97.5% CI 0.978–1.001), TT level (P = 0.147, 97.5% CI NA-0.998), APTT level (P = 0.049, 97.5% CI 0.836–1.004), INR level (P = 0.093, 97.5% CI 0.019–4.43E + 24), PT level (P = 0.154, 97.5% CI 0.010–2.026), WBC level (P = 0.031, 97.5% CI 1.264–41.178), NEU level (P = 0.022, 97.5% CI 0.014–0.679), LYM level (P = 0.046, 97.5% CI 0.015–0.907), CREA level (P = 0.039, 97.5% CI 0.960–0.999) and AST level (P = 0.170, 97.5% CI 0.999–1.012). The statistical characteristics of the model selected by stepwise (stepAIC) selection are summarized in Table 2. The following model was identified as the best predictive model (AIC = 260.86): 2.695843 − 0.63056 × (Jaundice = 1) − 1.08368 × (DM = 1) − 2.10445 × (Hepatitis = 1) + 1.152354 × (Pancreatic tumor = 1) + 1.071354 × (Bile duct tumor = 1) − 0.01185 × CA125 − 0.04929 × TT − 0.08826 × APTT + 26.03383 × INR − 1.9442 × PT + 1.979563 × WBC − 2.26868 × NEU − 2.0789 × LYM − 0.02038 × CREA + 0.00459 × AST. The ROC curve and leave-one-out method were used for the internal validation of the predictive model. The ROC curve was statistically significant (AUC = 0.7758) (Fig. 1). The validation result of the leave-one-out method showed that the accuracy of the model and the F measure were 0.887 and 0.939, respectively, with statistical significances. The nomogram was developed based on the predictive model (Fig. 2).

**Table 2:** Statistical characteristics of the stepwise (stepAIC) selected model for postoperative hemorrhage.

Variables	97.5% CI	z value	P
Jaundice	0.236–1.165	− 1.557	0.119
DM	0.146–0.807	− 2.513	0.012
Hepatitis	0.032–0.488	− 3.079	0.002
Pancreatic tumor	1.442–7.278	2.810	0.005
Bile duct tumor	1.136–8.338	2.127	0.033
CA125	0.978–1.001	− 2.006	0.039
TT	NA–0.998	− 1.449	0.147
APTT	0.836–1.004	− 1.964	0.049
INR	0.018–4.43E+24	1.667	0.093
PT	0.010–2.026	− 1.427	0.153
WBC	1.264–47.178	2.147	0.032
NEU	0.014–0.678	− 2.288	0.022
LYM	0.015–0.907	− 1.995	0.046
AST	0.999–1.212	1.370	0.171
CREA	0.960–0.999	− 2.058	0.039

*DM* diabetes mellitus.

## Discussion

Despite technical complexity and surgical morbidity associated with pancreaticoduodenectomy, LPD has been found superior to OPD in terms of feasibility, safety, and oncological results in several retrospective studies^3,18,19^. However, some studies have reported that LPD leads to more severe morbidity than OPD^20,21^. Predictive model based on regression analysis and the deep learning models have been developed for the early detection of clinical issues^22,23^. Besides modifying the surgical procedures of LPD^24–33^, the prediction of severe complications before the surgery can be useful for better preoperative preparation and for reducing the incidence of severe complications. The mortality rate of patients due to postoperative hemorrhage after LPD is higher than that due to other complications^21,34^. To our knowledge, this study is the first to report the risk-associated preoperative factors of LPD and to develop a predictive model and a nomogram of postoperative hemorrhage after LPD, and deep learning models may be potential good tools for improving the prediction performance in the future.

The predictive model required comprehensive preoperative data including admission symptoms, imaging examination, coexisting medical conditions, preoperative treatment, and blood tests (tumor markers, blood routine, liver function, renal function and coagulation function). We found that the preoperative data including coexisting medical conditions of DM and hepatitis were statistically related to the postoperative hemorrhage of LPD. DM and hepatitis are two major public health problems worldwide. The pancreatic fistula was the most common cause of postoperative hemorrhage, which required reoperation or interventional embolization. Some studies have reported that DM is related to pancreatic fistula after pancreatic surgeries^35–39^. Hepatitis was reported to be related to postoperative hemorrhage after liver transplantation^40^. Other risk-associated preoperative factors in our studies were tumor-related genomic background, immune microenvironment, tumor locations of pancreas and bile duct, level of tumor marker of CA125, coagulation function of APTT, INR, and PT levels, blood routine of WBC, NEU, and LYM levels^41–44^. Multivariant binary logistic regression and stepwise (stepAIC) selection were performed to develop the predictive model for postoperative hemorrhage of LPD. We internally validated the model using the ROC curve and leave-one-out method. The results of the internal validation were statistically significant. The nomogram based on the predictive model was developed for the easy use of the model.

This study has some limitations. First, the same LPD procedure was used for all the surgeries, and hence different procedures used at different medical centers were unexplored. Second, the sample size of the training cohort was relatively limited, and the external validation was not performed due to the difficulty to collect additional samples. Further studies are needed to accomplish the validation of the predictive model under a systems-level through integrating genomic and clinical information^45–48^, and explore the potential causal effects of these risk factors associated with postoperative hemorrhage under a Mendelian randomization framework^49–51^. As the LPD procedures were recommended by the Study Group of Pancreatic Surgery in the Chinese Society of Surgery of Chinese Medical Association^52^, our predictive model can be used only in patients who underwent LPD in the same surgical manner. Because of the high mortality caused by severe complications of LPD, studies are required to establish the predictive models for the other severe complications of LPD.

In conclusion, a relationship may exist between the postoperative hemorrhage and the coexisting medical conditions of DM and hepatitis. Postoperative hemorrhage after LPD can be predicted from the preoperative data of the patients. The predictive model with a practical nomogram can be used to estimate the risk degree of postoperative hemorrhage after LPD. Our nomogram will be useful for surgeons to minimize the postoperative complications of LPD by better preoperative preparation and communication with the patients’ families concerned with the intense situation between the doctors and the patients in China.
